# Stable expression of HIV-1 MPER extended epitope on the surface of the recombinant probiotic bacteria *Escherichia Coli* Nissle 1917 using CRISPR/Cas9

**DOI:** 10.1186/s12934-023-02290-0

**Published:** 2024-02-04

**Authors:** Nathaniel Ninyio, Katharina Schmitt, Gladys Sergon, Charlotta Nilsson, Sören Andersson, Nikolai Scherbak

**Affiliations:** 1 School of Medical Sciences, Faculty of Medicine and Health, Örebro University, Örebro, Sweden; 2 School of Science and Technology, Life Science Center, Örebro University, Örebro, Sweden; 3 Division of Clinical Microbiology, Department of Laboratory Medicine, Karolinska Institutet, Stockholm, Sweden; 4 Department of Microbiology, Public Health Agency of Sweden, Solna, Sweden; 5 Department of Public Health Analysis and Data Management, Unit for Vaccination Programmes, Public Health Agency of Sweden, Solna, Sweden; 6 Institute of Virology, Saarland University Medical Center, 66421 Homburg, Germany

**Keywords:** CRISPR/Cas9, HIV-1, Probiotic, Membrane-proximal external region (MPER), Outer membrane protein F (OmpF)

## Abstract

**Background:**

Mucosal vaccines have the potential to induce protective immune responses at the sites of infection. Applying CRISPR/Cas9 editing, we aimed to develop a probiotic-based vaccine candidate expressing the HIV-1 envelope membrane-proximal external region (MPER) on the surface of *E. coli* Nissle 1917.

**Results:**

The HIV-1 MPER epitope was successfully introduced in the porin OmpF of the *E. coli* Nissle 1917 (EcN-MPER) and the modification was stable over 30 passages of the recombinant bacteria on the DNA and protein level. Furthermore, the introduced epitope was recognized by a human anti-HIV-1 gp41 (2F5) antibody using both live and heat-killed EcN-MPER, and this antigenicity was also retained over 30 passages. Whole-cell dot blot suggested a stronger binding of anti-HIV-1 gp41 (2F5) to heat-killed EcN-MPER than their live counterpart. An outer membrane vesicle (OMV) – rich extract from EcN-MPER culture supernatant was equally antigenic to anti-HIV-1 gp41 antibody which suggests that the MPER antigen could be harboured in EcN-MPER OMVs. Using quantitative ELISA, we determined the amount of MPER produced by the modified EcN to be 14.3 µg/10^8^ cfu.

**Conclusions:**

The CRISPR/Cas9 technology was an effective method for establishment of recombinant EcN-MPER bacteria that was stable over many passages. The developed EcN-MPER clone was devoid of extraneous plasmids and antibiotic resistance genes which eliminates the risk of plasmid transfer to animal hosts, should this clone be used as a vaccine. Also, the EcN-MPER clone was recognised by anti-HIV-1 gp41 (2F5) both as live and heat-killed bacteria making it suitable for pre-clinical evaluation. Expression of OmpF on bacterial surfaces and released OMVs identifies it as a compelling candidate for recombinant epitope modification, enabling surface epitope presentation on both bacteria and OMVs. By applying the methods described in this study, we present a potential platform for cost-effective and rational vaccine antigen expression and administration, offering promising prospects for further research in the field of vaccine development.

## Background

The use of bacterial surface proteins for display of different bacterial or viral epitopes is an interesting and promising approach in vaccine development. The combination of candidate vaccine antigens and suitable bacterial surface proteins may form powerful structures for antigen-specific immune stimulation. Outer membrane proteins of *E. coli* play an important role in bacterial stress adaptation and intracellular metabolism, defining bacterial phenotypes [1]. The outer membrane (OM) of *E. coli* is one of the two lipid bilayers surrounding the bacterial cell. OM is asymmetric, with its outer leaflet largely composed of lipopolysaccharide (LPS) [2]. Additionally, the OM is associated with a variety of proteins including porins that are involved in a number of functions such as diffusion of ions and small molecules (OmpF), uptake of maltose (LamB), and host-defense (OmpX) [3]. In Gram negative bacteria, proteins comprise about 50% of the total OM mass [4].

OM proteins (OMPs) are porins that are becoming increasingly utilised in vaccine development research. This is because OMPs are recognised as pathogen-associated molecular patterns (PAMPs) by the pattern-recognition receptors of cells of the innate immune response, thereby making them highly immunogenic [5, 6]. Thus, they would have a potential as immunogens as such, but they are also used for adjuvant and immunomodulatory purposes.

Outer membrane vesicles (OMVs) are small (20-250 nm), spherical structures naturally produced by Gram-negative bacteria, including *E. coli*. These lipids-composed vesicles are derived from the bacterial cell envelope by budding [7]. OMVs contain various biomolecules, including proteins, lipopolysaccharides (LPS), and genetic material such as DNA and RNA. OMVs play a crucial role in bacterial communication, immune system evasion, and the delivery of virulence factors to host cells, making them a subject of significant interest in both bacterial pathogenesis research and potential biotechnological applications including vaccination [8].

The HIV-1 membrane-proximal external region (MPER) is an epitope of interest in HIV vaccine research. The MPER is about 24 amino acid residues long and it is an invariant region of the gp41 glycoprotein located within the HIV-1 envelope protein [9]. In addition to mediating viral entry into host cells, MPER also harbours epitopes of several well-characterised HIV-1 broadly neutralising antibodies (bNAbs) such as 2F5, 4E10, and 10E8 [9–12]. Expressing the MPER epitope on the surface of Gram-positive *Lactobacillus acidophilus* resulted in the activation of the immune system, predominantly eliciting Th1 and Th17 responses [13]. HIV-1 vaccine candidates generally aim at inducing a systemic immune response. Because HIV is transmitted via the mucosal route and impacts the gut-associated lymphoid tissues [14], an ideal HIV-1 vaccine should also induce an effective mucosal immune response.

Probiotic bacteria, such as gram-positive lactic acid bacteria, and gram-negative *Escherichia coli* Nissle 1917 (EcN), are able to colonise mucosal surfaces and recently they have been shown to be promising mucosal vaccine vectors of immunogenic epitopes [15–20]. In addition, probiotic bacteria may exhibit adjuvant and immunomodulatory properties [15, 16, 21, 22]. EcN has been shown to be immunomodulatory as it inhibits pathogenic bacterial invasion of epithelial cells [23], induces beta-defensin in epithelial cells [24], modulates T-cell proliferation [25], and elicits humoral B-cell responses [26]. The EcN probiotic bacteria may provide a platform for the expression of of candidate vaccine antigens.

The inclusion of the membrane milieu and surface presentation of immunogens would make it especially relevant for a HIV vaccine candidate [27]. The CRISPR/Cas9 gene editing system can be used for the insertion of heterologous sequences into bacterial chromosome with extremely high precision and efficiency [28, 29]. The use of CRISPR/Cas9 provides for the incorporation of a heterologous sequence into the bacterial chromosome with ultimate curing of CRISPR/Cas9-associated plasmids. It also eliminates the need to use associated antibiotics or supplements for plasmid maintenance.

Here, we describe the development of a stably transformed probiotic strain of *E. coli* Nissle 1917 that expresses the HIV-1 MPER fused to the outer membrane protein OmpF on the surface using the CRISPR/Cas9 editing system.

## Results

### Homology modelling of the recombinant OmpF-MPER protein

To identify appropriate locations for the MPER insert and guide the construct’s design, we conducted homology modeling. The pairwise protein sequence alignment between OmpF in Nissle and OmpF sequences from E. coli K-12 strains revealed a 99% identity. Consequently, the known crystal structure of the OmpF porin from E. coli K-12 serves as an excellent template for estimating the spatial position of the inserted MPER sequence.

**Fig. 1:**
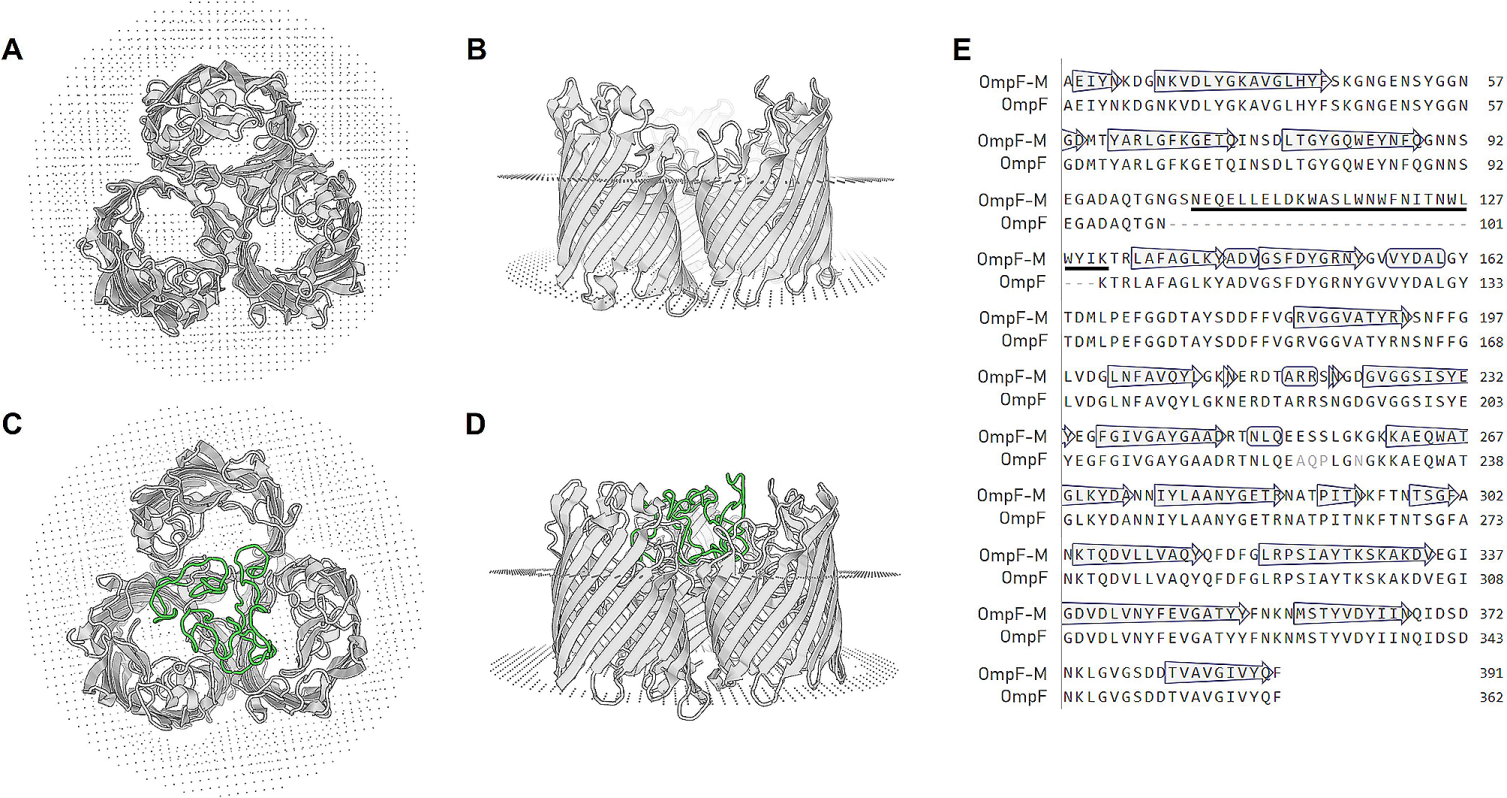
Homology modelling of the recombinant OmpF-MPER protein. Upper (**A**) and lateral (**B**) views of the OmpF protein trimer from *E. coli* K-12 strain (based on 6wtz.pdb). Upper (**C**) and lateral (**D**) views of the predicted OmpF-MPER protein from EcN-MPER, homology modelling is made on 6wtz.pdb structure using SWISS-MODEL tool. Green indicates location of the MPER sequence. (**E**) Sequence alignment of the OmpF-MPER (OmpF-M) chain A of the EcN-MPER to the OmpF K-12 (6wtz) template. MPER sequence is underlined in bold, arrows indicate transmembrane regions of the porin

Homology modelling of the OmpF-MPER protein indicated the positioning of the MPER epitope in the 3D structure of the individual OmpF proteins to be within the porin trimer (Fig. 1).

### Incorporation of the MPER epitope in the EcN genome

**Fig. 2:**
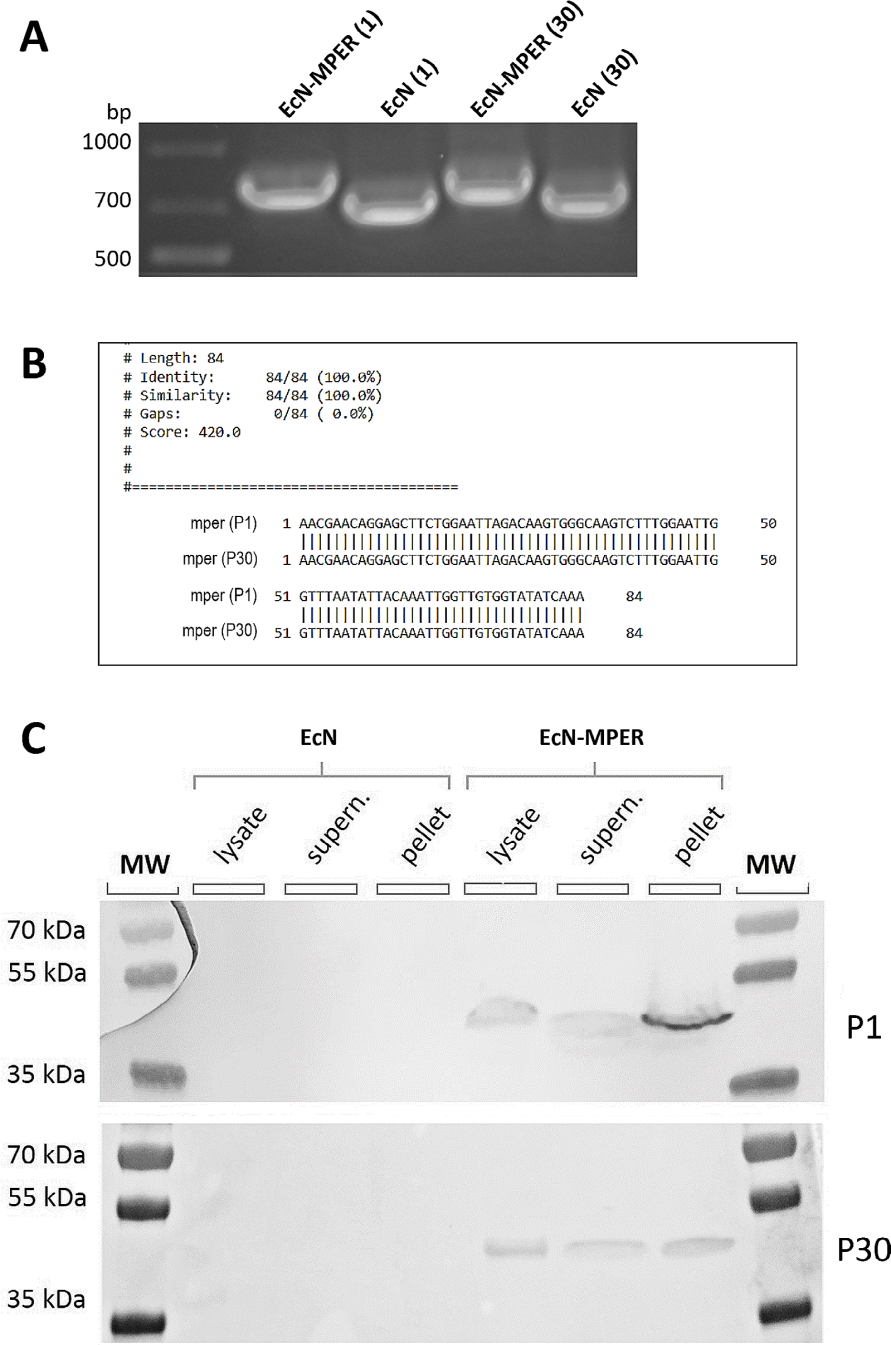
Analysis of the MPER insert stability over the passages. (**A**) PCR analysis for the presence of MPER insertion in the bacterial genome of the EcN. Higher (compared to EcN) molecular weight bands confirm the presence of the insert in the genome of EcN-MPER bacteria. (**B**) Pairwise sequence alignment of the sequenced MPER insert in passages 1 and 30 of the recombinant EcN-MPER. A sequence similarity of 100% was obtained through analysis with the Needle (EMBOSS) Global Alignment Tool, which connotes an absence of mutation. (**C**) Western blot to evaluate the expression of the MPER in EcN-MPER and non-recombinant EcN. The whole cell lysate (lysate), soluble fraction in the supernatant (supern.) and insoluble cell debris (pellet) of passages 1 and 30 (P1 and P30) were analysed after probing with 2F5 antibodies. The expected size of recombinant OmpF-MPER is 43 kDa

The presence of the MPER epitope in the recombinant EcN-MPER clone was verified by PCR using sequencing primers flanking the insertion position in the *ompF* gene. As seen in Fig. 2A, the PCR product from the EcN-MPER clone (passage 1) has a larger molecular weight (~ 840 bp) than the EcN wild-type lacking the MPER sequence with an amplicon size of ~ 750 bp, suggesting the presence of the additional 90 bp MPER sequence.

Sequencing confirmed seamless insertion of the MPER sequence in the desired chromosomal locus (Fig. 2B).

Successful expression of the MPER sequence was also confirmed by Western blot analysis of lysed EcN-MPER and EcN fractions. As shown in Fig. 2C, MPER in EcN-MPER fractions for passage 1 (P1) was recognised by the 2F5 antibodies. Western blot analysis showed a protein band of ~ 43 kDa, which corresponds to the expected size of the recombinant OmpF-MPER protein.

### MPER is expressed on the surface of the recombinant bacteria (EcN-MPER) and detected in an extracellular vesicle-rich fraction

**Fig. 3:**
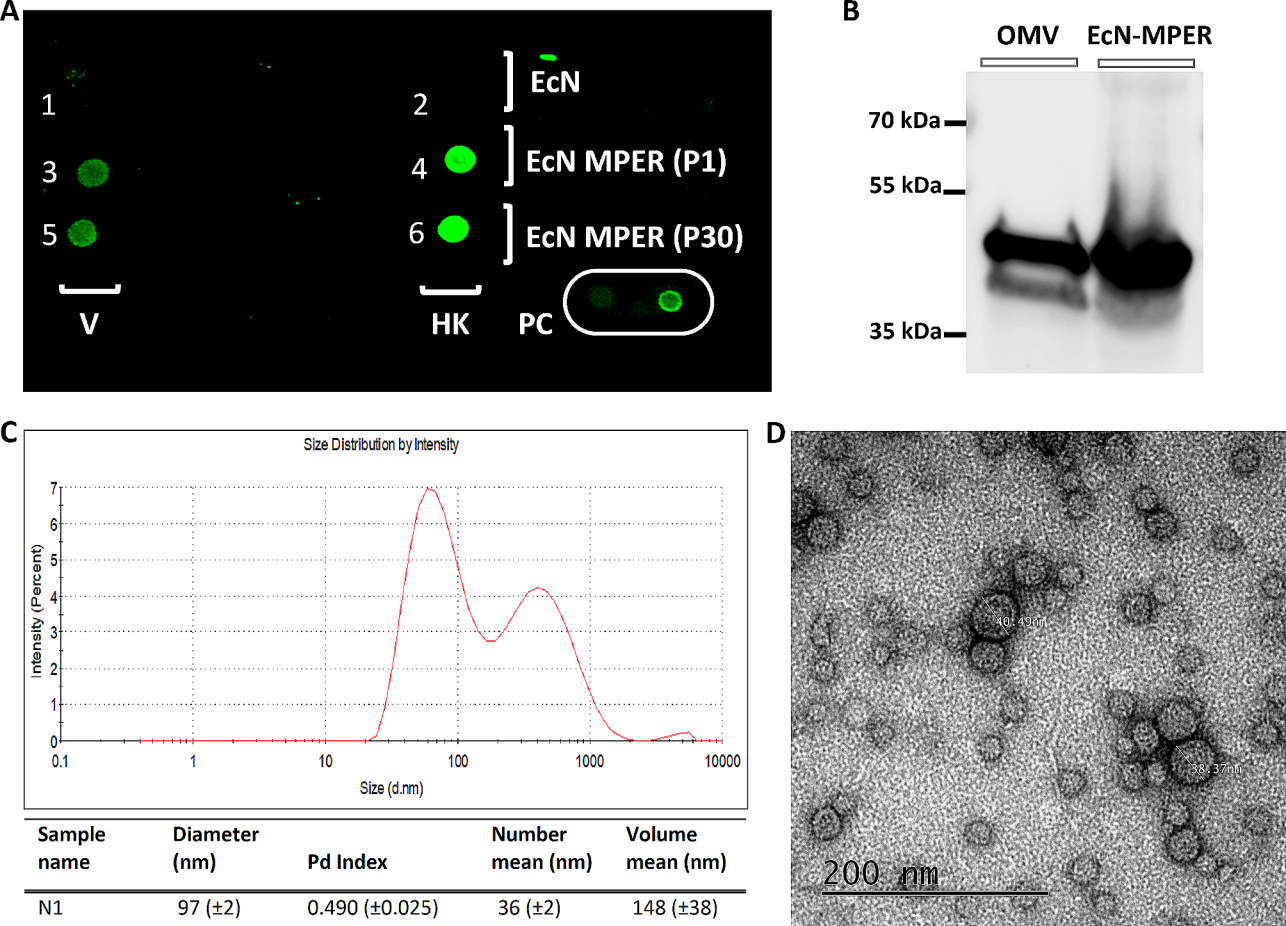
Whole cell dot bot and Western blot assay screening of surface expression of the MPER. (**A**) Dots representing whole cells immunostained with 2F5 primary antibodies of viable (V) or heat-killed (HK) EcN-MPER and EcN from passages 1 (P1) and 30 (P30) of the overnight cultures. The recombinant HIV-1 gp41 protein at concentrations of 15 and 150 ng was used as a positive control (PC). Detection was performed using the goat anti-human IgG IRDye® 800CW secondary antibody. (**B**) Western blot analysis of the outer membrane vesicles (OMVs) – rich fraction extracted from EcN-MPER culture supernatant. MPER was detected in the OMVs-rich fraction and positive control (EcN-MPER pellet) using HIV-1 gp41 (2F5) monoclonal antibody and rabbit anti-human HRP-conjugated IgG (Fc specific) secondary antibody. (**C**) DLS analysis of the OMVs fraction, and (**D**) negative stain TEM analysis of the same fraction

Since MPER was shown to be expressed, we proceeded with a whole bacterial cell dot blot to verify the presence of the epitope of interest on the cellular surface. In theory, CRISPR/Cas9 gene editing should have inserted the MPER oligonucleotide in the porin-gene, *ompF*, which is superficially harboured on the outer membrane surface of the EcN-MPER clone. This needed confirmation. Dot blots were performed on live and heat-killed EcN and EcN-MPER bacteria. An antibody labelling with human anti-HIV gp41 (2F5) monoclonal antibody demonstrated that both the viable (V) and heat-killed (HK) EcN-MPER clones were antigenic to the antibody (Fig. 3A). The immunofluorescence intensity of the EcN-MPER labelled with FITC-conjugated secondary antibody indicated stronger antigenicity to anti-HIV gp41 (2F5) in the heat-killed bacteria than in their live counterpart (Fig. 3A).

Having ascertained that MPER was surface-expressed on the EcN-MPER clone, we extracted an OMV–rich fraction from EcN-MPER culture supernatant using ultracentrifugation. We assayed this fraction using Western blot for the presence of MPER. The OMV-rich fraction was antigenic to the MPER-binding HIV-1 gp41 (2F5) monoclonal antibody (Fig. 3B) suggesting that MPER is harboured in OMVs produced by EcN-MPER. Prior to the ultracentrifugation, the culture supernatant, from which this OMV-rich fraction was extracted, underwent culturing on antibiotic-free LB agar to assess the presence of viable bacterial cells. No bacterial growth was observed after 24 h (data not shown).

To confirm the presence of outer membrane vesicles (OMVs) and evaluate their size in the fraction used for Western blot analysis, the OMV fraction underwent Dynamic Light Scattering (DLS). The calculation of OMV size originated from the conversion of the intensity-based diameter into the number-based diameter of the particles, producing two peaks in the Intensity vs. Size distribution diagram (Fig. 3C). Additionally, we performed direct imaging using transmission electron microscopy (TEM) to obtain the topographical size of particles. Negative stain TEM shows an abundance of OMVs (Fig. 3D) in the sample with average sizes smaller than those indicated by DLS. The size distribution of OMVs ranged approximately between 15 and 75 nm in diameter (estimated visually). TEM also revealed the presence of free OMVs and aggregated with OMVs fragments of bacterial flagella and fimbriae in the sample (data not shown).

### Quantification of MPER expression by the EcN-MPER clone

**Fig. 4:**
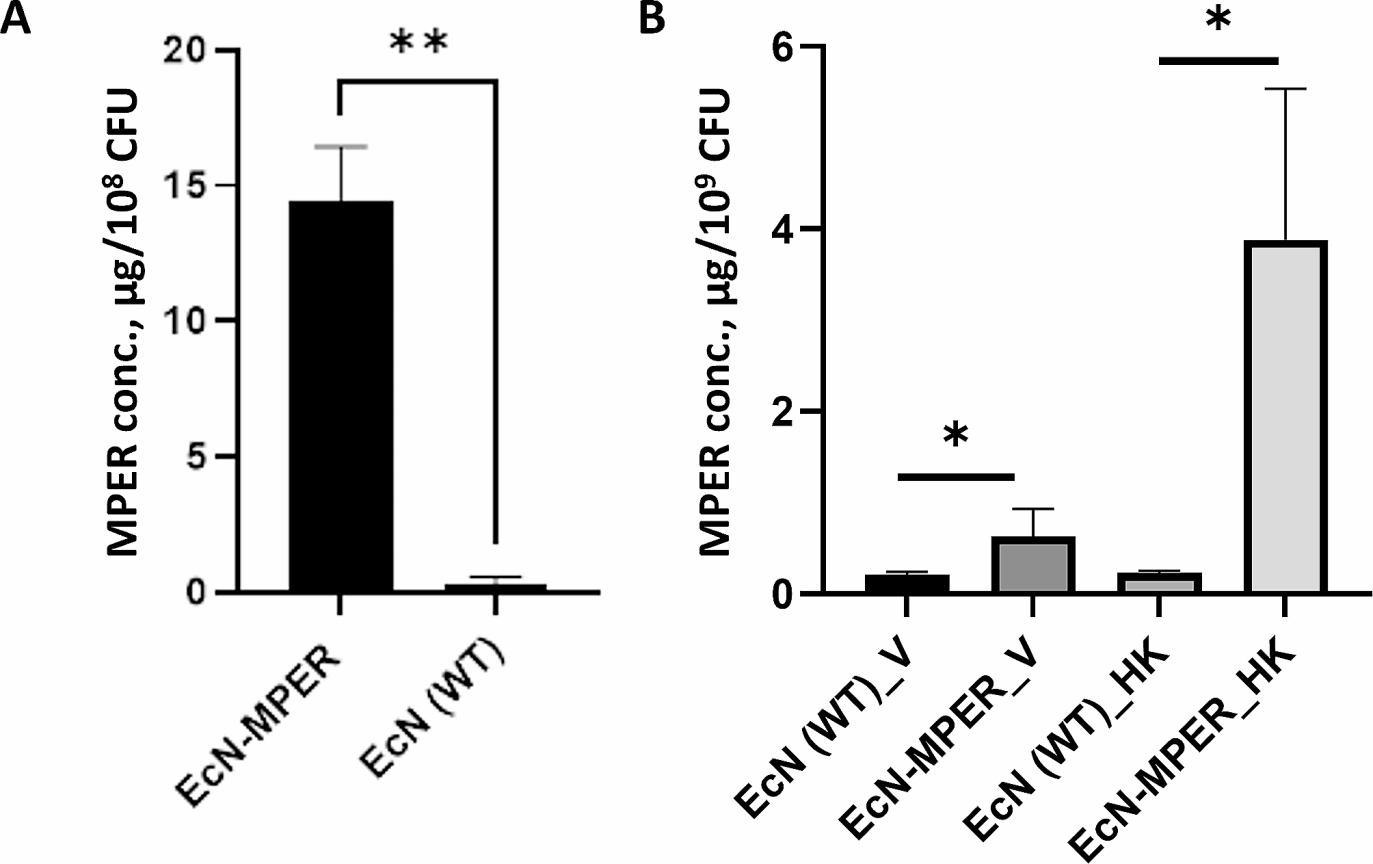
Quantification of MPER concentration in bacteria via indirect ELISA. (**A**) From recombinant (EcN-MPER) and non-modified (EcN) bacterial lysates. The antigen concentration was determined using a standard curve plotted with absorbance readings applying a serially diluted HIV-1 gp41 antigen. (**B**) Quantitation of surface-expressed MPER through a whole cell ELISA of non-modified EcN and recombinant EcN-MPER from combined passages 1, 15, 20, 25 and 30. Samples with viable bacteria are marked with V and with heat-killed – HK. Bars are shown with mean and standard deviation of the 5 different passages. Comparative analysis was performed using (**A**) Welch’s t test or (**B**) Mann Whitney test; where *p < 0.05 and **p < 0.005

After having confirmed the expression of the MPER in bacterial lysate (Fig. 2C) and on the bacterial surface (Fig. 3A), we quantified the MPER expression. The total amount of MPER produced in the EcN-MPER lysate was quantified by indirect ELISA and found to be 14.3 ± 2,01 µg/10^8^ CFU (Fig. 4A) (0.143 pg/CFU).

Since the recognition of the epitopes by the antibodies is influenced by the epitope’s accessibility, the antigenicity of both live and heat-killed whole bacteria was studied by ELISA. As shown in Fig. 4B, antigenicity depended on the state of the bacteria. MPER-expression was quantified to be ~ 0.07 µg/10^8^ CFU (or 0.0007 pg/CFU) in viable bacteria and ~ 0.4 µg/10^8^ CFU (or 0.004 pg/CFU) in heat-killed EcN-MPER.

## Discussion

In this study, we used CRISPR/Cas9 editing to produce a recombinant probiotic strain of *E. coli* Nissle 1917 (EcN) that constitutively expressed the HIV-1 epitope MPER as part of the OmpF bacterial porin. We report a stable incorporation into the bacterial genome for at least 30 passages and the ability of the 2F5 neutralising antibody to recognise the corresponding viral epitope on the bacterial surface. The genomic integration of the epitope provides the added benefit of eliminating the need of a plasmid-originated antibiotic resistance gene in the recombinant bacteria. This decreases the risk of antibiotic resistance gene transfer to a host, should EcN-MPER or other candidate vaccine constructs be used as a live probiotic vaccine based on similar principles [30]. Previously CRISPR/Cas9 modification of the *ompF* loci was shown to be effective for the surface presentation of the hepatitis B virus (HBV) S antigen and human papilloma virus (HPV) L2 protein in *E. coli* strains ER0808 and ER2566 [31], but to our knowledge this is the first example of the use of CRISPR/Cas9 gene editing to express potential vaccine antigens in a probiotic strain.

Among OMPs, the outer membrane protein F (OmpF) and C (OmpC) are the two most common porins that make up 2% of the total cellular proteins, and OmpF is the most structurally and functionally characterised [3, 32, 33]. It was demonstrated that recombinant OmpF protein from *E. coli* could stimulate strong immunoglobulin G (IgG) antibody responses and provided high protection against lethal infection in mice with the highly virulent *E. coli* strain PCN033 [32]. Furthermore, it was also shown that the OmpF of *E. coli* can be successfully used for surface presentation of viral epitopes [31]. Here, homology modelling of the OmpF protein indicated a position of the MPER epitope in the 3-D structure of the individual OmpF proteins within the porin trimer. Insertion of the epitope elongates the extracellular loop 2 of the porin, however for the exact secondary structure of inserted amino acids in the extracellular space, additional protein crystallography studies should be made. A repeated display of viral epitopes has proven to be pivotal in stimulating the immune system response [34]. The close proximity of three MPER epitopes within the OmpF protein trimer lead to a multimeric presentation of the HIV epitopes on the recombinant bacteria’s surface. This concept is supported by the observation that the outer membrane proteins, with OmpF being a prominent porin, form a closely interconnected network on the surface of E. coli [35].

After showing that the EcN clone contained the MPER sequence, we studied the stability of the insertion. Since presence of the viral MPER sequence is naturally abnormal for the EcN genome, we studied if subsequent passaging during a 30-day period would lead to exclusion or mutation of the inserted sequence. Assuming that the EcN has a 20 min doubling time under unlimited [36] conditions and 24 h culture time per passage, it is possible to approximate that each cell undergoes approximately 72 divisions per passage and over 2100 divisions during 30 passages. Sequencing showed that the CRISPR/Cas9 method is able to maintain a stable MPER-insertion over the numerous generations of the recombinant bacteria. Western blot and ELISA analyses demonstrated that the MPER epitope was recognised in the bacterial lysate by the human anti-HIV-1 gp41 (2F5) monoclonal antibody even after multiple passaging of the bacterial culture.

In whole cell ELISA and dot blot analyses we confirmed the surface expression and accessibility to the neutralising antibody of the MPER epitope. Surface display of antigens facilitates their interaction with players of innate and acquired immunity, such as dendritic cells, monocytes, and macrophages [37]. Thus, the enrichment of the OmpF-MPER on the 6 µm^2^ cell surface area of the EcN may provide more room for interaction and recognition of the epitope by pattern recognition receptors (PRRs) on the aforementioned immune cells [38].

Although the mechanism underlying the immunogenicity of probiotics is known to be strain-specific, there is increasing evidence that suggests the occurrence of a cross talk between probiotic-produced outer membrane vesicles and mucosal epithelial cells [39, 40]. Outer membrane vesicles (OMVs) are constitutively produced extracellular vesicles that originate from the outer membrane (OM) of Gram-negative bacteria [41]. The size of the OMVs is reported to be in range between 10 and 300 nm in diameter [42]. Our Dynamic Light Scattering (DLS) analysis of the OMVs fraction indicated a polydisperse nature of the sample with two peaks (Fig. 3C). One peak exhibited a particle size ranging from approximately 20 to 110 nm in diameter, corresponding to the expected size of the OMVs. The average diameter of OMVs in the sample, calculated by the DLS analytical software, was 97 ± 2 nm, which is higher than visually observed in the TEM images. This discrepancy can be explained by the polydisperse nature of the sample and the presence of larger structures, which may affect size determination [43]. The second peak suggests the presence of high molecular weight aggregated species [44], and aggregates of the OMVs were also evident in the TEM analysis. Additionally, TEM analysis of the OMVs’ fraction revealed the presence of broken pieces of flagella that were associated with the aggregates of the OMVs.

OMVs encapsulate material of cytoplasmic and periplasmic origin, like DNA, RNA or proteins. However, the exact composition of the OMVs varies between different species and strains of bacteria. It was also shown that OMVs from *E. coli* incorporate approximately 1% of their outer membrane material including 0.2–0.5% of the OM and periplasmic proteins [42]. Proteomic analysis of the OMVs from *E. coli* Nissle in the study by Aguilera et al. revealed the presence of a large repertoire of different proteins contributing to survival of bacteria and host colonisation [45]. Identification of OmpF proteins among others in that study prompted us to investigate the presence of MPER in the OMVs fraction. In the present study, the MPER DNA sequence was incorporated into the *ompF* genomic locus of EcN, it was therefore natural to observe binding of the MPER-specific 2F5 antibodies to the purified OMVs. Presentation of the viral epitopes on the surface of the OMVs is an interesting approach for future vaccine development [46].

We have observed a higher affinity of the 2F5 antibodies to heat-killed EcN-MPER compared to live EcN-MPER in the whole cell dot blot. The MPER epitope adopts a helical structure and correct orientation of the amino acids is crucial for the antibody recognition. In the gp41 protein of the HIV-1 the MPER epitope is situated in close proximity to the cell membrane where hydrophobic interactions between amino acid residues of the MPER and the cell membrane are guaranteeing the proper orientation of the amino acids that are important for the interaction with the neutralising antibodies. It can be hypothesised that the orientation of the epitope in the OmpF-MPER chimeric protein was not optimal for the antibody recognition due to some interactions within the porin trimer, while denaturation of the protein after heat-killing of the bacteria made the epitope more accessible. One potential way to optimize antibody recognition of the epitope is to stabilize the epitope structure using a variety of polypeptide linkers [47].

Another interesting question is to what extent the MPER epitope is present on the surface of the EcN. Based on our ELISA analysis, we estimated that the surface of each colony-forming unit (CFU) of EcN contained approximately 0.0007 pg of OmpF-MPER under native conditions, and about 0.004 pg in the case of heat-killed EcN-MPER bacteria. Knowing the molecular weight of the OmpF-MPER protein it is possible to approximate that this corresponds to roughly 56,000 OmpF-MPER monomers expressed at the surface of each CFU of the heat-killed bacteria. Notably, quantification under native conditions yielded an approximately six-fold lower value per CFU compared to heat-killed EcN-MPER. By utilising data on the cell surface area occupied by each porin and the total surface area of the bacteria, an estimation was previously made that *E. coli* contains approximately 10^5^ porins [48]. The quantities of porin observed in this study closely resemble those values. However, it cannot be entirely ruled out that the presence of potential cytoplasmic proteins in heat-killed bacteria may have influenced the quantification in this study. Surface presentation of the antigens have been suggested to be a useful strategy for the induction of humoral and mucosal immune responses to antigens [49]. The advantage of using a probiotic *E. coli* strain Nissle 1917 lies in the well documented safety of the strain [50]. Probiotics offer notable advantages in terms of safety when administered to different groups of patients, however in immunocompromised patients, neonates and hospitalised patients probiotics should be used with caution [51]. Thermal inactivation has been evaluated and has been found to address the safety aspect such as the potential risk of antibiotic gene transfer, microbial translocation and supply chain requirements associated with the pharmaceutical properties of live vaccines [52].

This study evaluated the antigen display properties of live and heat-killed EcN-MPER. Both the immunoblot and whole cell ELISA demonstrated that heat killing enhanced the binding of EcN-MPER to human anti-HIV-1 gp41 (2F5). This is probably because the heating facilitated the exposure of the antigens [53], through e.g. conformational changes and release of internal antigens not expressed on the cell surface. Comparative studies into the effectiveness of live and heat-killed forms of *E. coli* Nissle 1917 have demonstrated that both were similarly effective in the stimulation of the colonic epithelial chemical defense system through induction of the antimicrobial peptide hBD-2 [54] and comparable in the therapeutic treatment of acute and chronic colitis [55].

There are advantages and disadvantages of using live or killed bacteria for immunisation. The possibility of utilising the potential of probiotics to colonise for a while might be an advantage, as compared to using heat-killed bacteria. You thereby get a longer exposure of the antigens to the mucosal surface, i.e. to the mucosal immune system, as well as a more pronounced adjuvantic stimulation. Utilising inactivated and/or lysed bacteria presents advantages in terms of antigen dosage standardization and the potential mitigation of risks associated with prolonged colonization, if deemed necessary. When preparing for studies in animals or humans, this should be kept in mind.

## Conclusion

We successfully inserted HIV-1 gp41 MPER into the outer-membrane protein OmpF of *E. coli* Nissle 1917 through CRISPR/Cas9 editing. The developed EcN-MPER clone was shown to be genetically stable over many passages. By using CRISPR/Cas9 editing, the clone is devoid of extraneous plasmids and antibiotic resistance genes. Furthermore, it was shown to be antigenic to anti-HIV-1 gp41 (2F5) both as live and heat-killed bacteria making it suitable for pre-clinical evaluation. The presence of OmpF on the surface of the bacteria and the OMVs released from the surface makes this protein an interesting candidate for modification with recombinant epitopes, facilitating surface presentation of the epitopes on both bacteria and OMVs. The EcN-MPER as such is suggested as a novel probiotic HIV-1 vaccine candidate for further assessment. Moreover, by applying the methods described in this study, a potential platform is revealed for cost-effective and rational vaccine antigen expression and administration, offering promising prospects for further research in the field of vaccine development.

## Methods

### Identification of the insertion locus for MPER

We used the crystal structure for the OmpF protein (6wtz.pdb, OmpF porin from *E. coli* K-12) from the Protein Data Base (https://www.rcsb.org/) to identify the potential position for the insertion of the MPER (ELLELDKWASLWNWFNITNWLWYIK) in the extracellular loop 2 of the porin. Through homological alignment, we determined the corresponding region in the EcN OmpF protein and thereafter, in the *ompF* gene of *E. coli* Nissle 1917 (GenBank: CP007799.1, RefSeq:WP_000977927.1). This information guided our strategy for the determination of the sequences for gRNA and homologous arms of the ompF-MPER oligonucleotide template (see below). To estimate the impact of the insert on the structure of the OmpF we have used the online SWISS-MODEL tool (https://swissmodel.expasy.org/) to build a homology model of the recombinant OmpF-MPER protein. The 6wtz.pdb protein structure file for OmpF was used as a template for that purpose.

### Plasmid construction

**Fig. 5:**
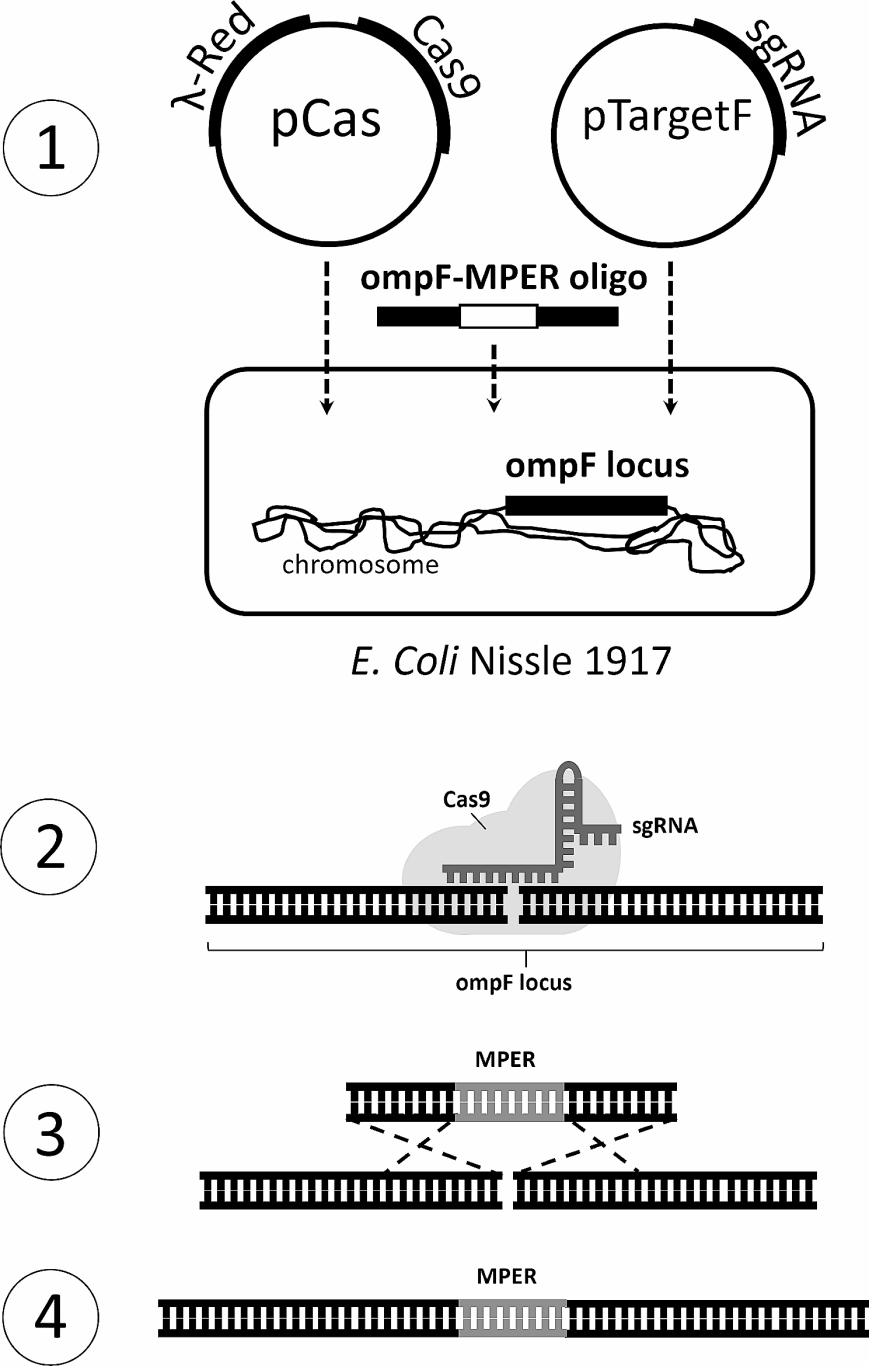
CRISPR/Cas9-mediated editing of the EcN genome with MPER insert. A schematic representation of the development of the EcN-MPER clones using CRISPR/Cas9 according to [29] with modifications. (1) Transformation of EcN with plasmids and the template oligo; (2) Nuclease Cas9 is guided by gRNA to create a site-specific break in bacterial gDNA; (3) OmpF-MPER oligonucleotide is used as the template for gDNA repair through homologous recombination; (4) PCR and sequencing to confirm the insertion of MPER in the desired position inside ompF gene

CRISPR/Cas9 editing of the *E. coli* Nissle 1917 (EcN) was done according to the protocol described in [29]. A schematic overview is given in Fig. 5. Plasmids pCas (Addgene plasmid # 62,225) and pTargetF (Addgene plasmid # 62,226) were a gift from Dr Sheng Yang (Shanghai Institutes for Biological Sciences). The plasmids, which were harboured in *E. coli* DH5α, were purified using the alkaline lysis method [38]. Then, guide RNA (gRNA) design was performed using the online tool CHOPCHOP (https://chopchop.cbu.uib.no/) [56]. This gRNA is designed to assist in targeting the double-strand break in the desired locus of chromosomal DNA. The sequence corresponding to the gRNA was introduced in pTargetF by PCR with a customised primer pair, pTF_ompF107-F and pTF_ompF107-R (Table 1) using Phusion High-Fidelity DNA polymerase (ThermoFisher Scientific, USA). Consequently, self-circularisation of the obtained PCR product was achieved using T4 ligase (ThermoFisher Scientific, USA) according to the manufacturer’s protocol. The correct insertion of gRNA sequence in the pTargetF plasmid was verified by sequencing. The template oligo was synthesised by GenScript Biotech, USA, and delivered as a part of the pUC57 plasmid. The OmpF-MPER insert comprises the MPER sequence flanked by two sequences homologous to *ompF*, each spanning 300 bp upstream and downstream of the insertion site at EcN *ompF* chromosomal locus. The insert was excised from pUC57 by EcoRV digestion, separated by DNA electrophoresis and purified from the gel using a QIAquick gel extraction kit (Qiagen, Germany). The obtained oligo was used as a template for the λ-Red recombinase for the insertion of the MPER epitope into bacterial genome.

**Table 1:** Plasmids, primers and oligo used in the current study

*Plasmids and primers*	*Characteristics*	*Source*
*Plasmids*		
pCas	Kanamycin resistance Temperature sensitive: cultured at 30 °C, Size: 12,545 bp	[22], Addgene plasmid #62,226
pTargetF	Spectinomycin resistance, Size: 2117 bp	[22], Addgene plasmid #62,226
pTargetF-OmpF	Modified pTargetF; 20 bp change of gRNA	This study
pUC57-ompF-MPER	Plasmid vector containing MPER surrounded with 300 bp (from each side) homologous to *ompF* sequences.	GenScript
***Primers***		
pTF_ompF107-F	CGCGTCGTTTTAGAGCTAGAAATAGC (Tm^1^ = 66.5^o^C) Insertion of gRNA into pTargetF	This study
pTF_ompF107-R	TTTTGTTACCAGTTACTAGTATTATACCTAGGACTGAGTC (Tm^1^ = 66.5^o^C) Insertion of gRNA into pTargetF	This study
118 F Pre-OmpF forward primer)	GTAGCACTTTCACGGTAGCG (Tm^1^ = 60 ºC) Binds to the plus strand upstream of *ompF*	This study
105R2 (OmpF reverse primer)	GTAGCTGATAGAACCGCCAACAC (Tm^1^ = 60 ºC) Binds to the minus strand of *ompF*, downstream the insertion	This study
108 F (pTargetF forward primer)	TTTCCTGCGTTATCCCCTGA (Tm^1^ = 60ºC) Binds to the plus strand of pTargetF, upstream the sgRNA	This study
108R (pTargetF reverse primer)	CGACGCGTTTTGTTACCAGT (Tm1 = 60 ºC) Binds to the minus strand of pTargetF-OmpF and is specific for the OmpF-gRNA	This study
109 F (OmpF forward primer)	AACAGTTACGGTGGCAATGG (Tm1 = 60 ºC) Binds to the plus strand of *ompF*, upstream the insertion	This study
109R (MPER reverse primer)	CCAAAGACTTGCCCACTTGT (Tm1 = 60 ºC) Binds to the minus stand of MPER	This study

^1^ Tm is the primer melting temperature used during PCR.

### Transformation of bacteria

EcN bacteria were a gift from Ardeypharm GmbH (Germany). Bacteria were transformed in two rounds, first with the pCas plasmid, and then with the mixture of the pTargetF-OmpF plasmid and ompF-MPER oligonucleotide. Prepared by a standard protocol [57], electrocompetent EcN were electroporated with pCas in 1 mm electroporation cuvettes at 1.8 kV, 200 Ω and 25 µF using a GenePulser (Bio-Rad, USA). After electroporation, transformants were recovered in SOC medium for 1 h at 30ºC. This was followed by overnight incubation on Luria-Bertani broth (LB, Lennox formulation; Becton, Dickinson U.K.) agar plates supplemented with kanamycin (30 µg/ml). Then, pCas-containing clones of EcN (EcN-pCas) were verified by colony PCR and made electrocompetent as described above. Expression of λ-Red in EcN-pCas clone was induced by the addition of arabinose (10 mM final concentration) to the bacterial culture. Subsequently, EcN-pCas (40 µl) were transformed with a mixture of pTargetF-ompF (100 ng) and template oligo ompF-MPER (400 ng) at 1.8 kV, 200 Ω and 25 µF. Transformants were recovered in SOC medium and cultivated on LB agar plates supplemented with kanamycin (30 µg/ml) and spectinomycin (50 µg/ml) at 30 °C overnight. Plasmid curing was done as described in [29] which resulted in stably transformed clones named EcN-MPER. Colony PCR and DNA sequencing were performed to verify the genomic presence and the position of the insert in the EcN-MPER clone. These were performed using *ompF*-targeting primers (pTar_Omp-108 F and pTar_Omp-108R or MPER_Omp-109 F and MPER_Omp-109R, Table 1).

### Genetic stability assessment of the MPER insert

To assess the stability of the MPER sequence insert in the EcN-MPER clone, 30 passages of the EcN-MPER were prepared. Briefly, the aliquot of the primary − 80^o^C glycerol stock culture of EcN-MPER clone was inoculated on antibiotic-free LB agar and incubated overnight at 37 °C. A single colony was then inoculated in LB broth and incubated overnight at 37 °C with shaking at 220 rpm (final OD_600_ ≤ 1.2). This culture was labelled “Passage 1”. Passage 1 was inoculated into LB broth in a 1:10 000 dilution and incubated at 37 °C and at 220 rpm overnight (final OD_600_ ≤ 1.2); this was labelled “Passage 2”. The passaging was repeated until passage 30 was obtained. Aliquots of different passages were saved for the PCR and protein immunoblot analysis. PCR was done using primers MPER-Omp109F and MPER-Omp109R which are specific to the bacterial chromosome flanking the MPER insertion site. The amplicons were separated on 1% agarose gel, excised and purified using QIAquick gel extraction kit (Qiagen, Germany). These purified PCR amplicons from passage 1 and passage 30 were subjected to DNA sequencing to check for any mutations in the MPER gene. The MPER gene sequences for both passages, as determined by DNA sequencing, were compared using the Needle (EMBOSS) Global Alignment Tool (https://ebi.ac.uk/Tools/psa/emboss_needle/).

### Dot blot and Western blot analysis for the verification of epitope expression

To confirm the expression of the MPER polypeptide on the surface of EcN and to evaluate the stability of such expression after several passages, a dot blot analysis was performed as previously described by Chamcha et al. [58] with a few modifications. Both the overnight culture and cultures grown to exponential phase were evaluated for MPER expression. Briefly, 10 µl of the subculture of wildtype EcN and the recombinant EcN bacteria harbouring OmpF-MPER from the 1st and 30th passages from the overnight culture were pelleted by centrifugation. The pellet was washed twice with phosphate buffered saline containing 0.1% Tween 20 (PBST) and then resuspended to the original volume with PBST. Heat-killed bacteria were obtained by heating a bacterial suspension in PBS at 60 °C for 1 h. Bacterial suspensions were transferred to a nitrocellulose membrane and left to air dry for 1 h. The membrane was blocked with 5% skimmed milk (w/v in PBST) and probed with a 1:5 000 dilution of human anti-HIV-1 gp41 (2F5) monoclonal antibody (NIBSC, United Kingdom). The 2F5 antibody recognises the ELDKWA epitope of the MPER. Washing with PBS was repeated three times. After the washing step, the membrane was probed with goat anti-human IRDye®800CW secondary antibody (LI-COR, USA) at a 1:15 000 dilution for 1 h. Fluorescent detection was performed using the Odyssey Fc imaging system (LI-COR, USA).

Expression of the MPER in the lysates of the EcN-MPER (passages 1 and 30) was verified with Western blot using a 1:5 000 dilution of human anti-HIV-1 gp41 (2F5) monoclonal antibody (NIBSC, United Kingdom) and 1:7 500 dilution of rabbit anti-human HRP-conjugated IgG (Fc specific) antibody (Sigma-Aldrich, USA).

### Purification of outer membrane vesicles from EcN-MPER

Following confirmation of MPER expression on the surface of EcN-MPER clones, we analysed if MPER could be detected in outer membrane vesicle (OMV) – rich fractions isolated from EcN-MPER culture supernatant. Slightly modifying the method previously described by Wei et al. [59], a single colony of EcN-MPER from an overnight culture on LB agar was inoculated into 10 mL of LB broth. This was followed by overnight incubation at 37 °C with shaking at 220 rpm. This was inoculated into 1 L of LB broth and incubation was continued at 37 °C with shaking at 220 rpm. Culture centrifugation was performed at 4ºC and 5 000 *× g* for 15 min. The supernatant was transferred to clean tubes and the centrifugation was repeated at 4ºC and 15 000 *× g* for 15 min. Next, filtration of the supernatant was performed using 0.45 μm syringe filters. To confirm that the filtrate was devoid of bacterial contamination, 100 µL of the filtrate was inoculated on antibiotic-free LB agar and incubated for 24 h at 37 °C.

Furthermore, the cell-free filtrate was concentrated using centrifugal concentration tubes with a 10 kDa molecular weight cut-off. The filtrate was 50× concentrated at 3 000 *× g* and at + 4 °C. Next, ultracentrifugation was performed at 160 000 *× g* for 5 h and the supernatant was carefully aspirated leaving the OMV-rich pellet undisturbed. The pellet was washed by the addition of filter-sterilised PBS followed by ultracentrifugation at 160 000 *× g* for 2 h. Finally, the supernatant from the wash step was aspirated and the pellet was dissolved in 500 µL of PBS. To determine if this OMV-rich fraction contained MPER, a Western blot was performed using human anti-HIV-1 gp41 (2F5) monoclonal antibody (NIBSC, United Kingdom) and rabbit anti-human HRP-conjugated IgG (Fc specific) secondary antibody (Sigma-Aldrich, USA). EcN-MPER pellet was used as a positive control. The OMV-rich fraction was stored at -80 °C.

### Viability check and colony forming unit count

Colony forming unit (CFU) counting was performed on live cells of the EcN and EcN-MPER passages 1 and 30. For a colony-forming unit count, serial 10-fold dilutions of bacterial suspension were plated out on LB agar plates in triplicates followed by overnight incubation at 37 °C. Viability control of heat-killed cells was carried out as detailed in [60]. Briefly, 1 ml of the bacterial suspension at exponential phase was centrifuged and washed three times in PBS. The viable cells were thereafter resuspended to the original volume using PBS and heat-killing was performed at 65^o^C for 1 h before the cell suspension was plated out on LB agar plates.

### Indirect ELISA for quantification of total antigen in cell lysates

A standard curve was prepared with the recombinant HIV-1 M gp41 (Jena Bioscience, Jena, Germany) using 0.5, 1, 2, 5, 10 and 15 µg/ml dilutions of the protein. Then 100 µL of each dilution was loaded into the respective wells in a 96-well plate (ThermoFisher Scientific, USA). Standards were loaded in triplicates. Also, an aliquot of EcN-MPER bacteria from an overnight culture (O.D. ≤ 1.2) was subjected to a CFU (colony forming unit) count as described above. The rest of the overnight culture was pelleted by centrifugation and was resuspended in lysis buffer (25 mM HEPES, 500 mM NaCl; 2 mM phenylmethylsulphonyl fluoride (PMSF), 10 mg/ml lysozyme, pH 7.4) in a volume equivalent to the initial culture volume. Lysis of bacteria was facilitated by sonication on ice at 200 Hz for 20 s for 10 cycles. Then, 100 µL of the lysate was loaded into a 96-well plate (ThermoFisher Scientific, USA) in triplicates. Wildtype EcN grown and lysed as described above was used as the negative control. The wells were coated overnight at 4 °C and then washed three times with TBST (50 mM Tris-HCl, 150 mM NaCl, 0.1% (v/v) Tween 20; pH 7.4). The wells were blocked with 1% bovine serum albumin (BSA) at room temperature for 1 h and washed thrice in TBST. Furthermore, a 1:5 000 dilution of human anti-HIV-1 gp41 2F5 monoclonal antibody (NIBSC, United Kingdom) in TBS was added to each well and incubated at room temperature for 2 h. After three washes with TBST, HRP-conjugated rabbit anti-human monoclonal secondary antibody (Sigma-Aldrich, USA) was added at a 1:5 000 dilution to the wells and incubated additionally for 2 h. The wells were washed again with TBST and colour development was performed for 25 min using TMB substrate kit (ThermoFisher Scientific, USA). The process was stopped with 2 M sulphuric acid. Absorbance readings were measured at 450 nm with a microplate reader (BMG Labtech, Germany). The unknown MPER concentration was calculated from the equation of a straight line which was derived from the absorbance values of the serially diluted HIV-1 M gp41 standard.

### Whole cell ELISA for quantification of surface-expressed antigen

A whole cell ELISA was performed to quantify the surface-expressed MPER both on live and heat killed cells. The protocol as described in [61] was followed albeit with a few modifications. Cultures of the EcN and CRISPR/Cas9-modified EcN from passage 1, 15, 20, 25 and 30 were grown as described above and aliquots were taken from both the overnight cultures and from the subcultures grown to OD_600_ of 0.6. The culture media was discarded after centrifugation at 17 000 *×* g, and cells were washed twice with PBS pH 7.4 and reconstituted to the original volume. Aliquots representing the EcN and the various passages of the EcN-MPER were thereafter apportioned out for heat inactivation at 65^0^C for 1 h. The bacterial suspension (100 µl) from the EcN and the various EcN-MPER both from live and heat killed versions, was added to MaxiSorp™ 96 well plate (Thermofisher Scientific, USA) and left to incubate overnight at 37 °C. The HIV-1 gp41 (537–811) recombinant protein (Jena Bioscience, Germany) was used to generate a standard curve ranging from 14 000 pg/ml to 0.01 pg/ml and concomitantly served as the positive control.

ELISA washing steps were done three times with PBST (pH 7.4) and blocking was achieved with 5% non-fat milk in PBST for 2 h at room temperature. The plate was washed as above and wells were treated with 100 µl (per well) of the human anti-HIV-1 gp41 2F5 monoclonal antibody (NIBSC, United Kingdom) at a 1:5 000 dilution for 2 h. The plate was washed with PBST before adding the rabbit antihuman horseradish peroxidase secondary antibody (Sigma-Aldrich, USA) at a dilution of 1:5 000. Incubation was carried out at room temperature for 2 h and after the washing step (as above). TMB substrate (Thermofisher Scientific, USA) was added followed by incubation for 25 min. The enzymatic reaction was stopped by adding 2 M sulphuric acid. Absorbance readings were done using the Fluostar Omega microplate reader at 450 nm (BMG Labtech, Germany).

### Dynamic light scattering and transmission electron microscopy

Analysis of samples containing outer membrane vesicles by dynamic light scattering (DLS) was performed using commercial service at SOLVE analytical laboratory (Lund, Sweden). OMV containing samples were analysed using Zetasizer Nano ZS instrument (Malvern, UK) and Zetasizer Software 8.01 (Malvern, UK). Transmission electron microscopy (TEM) was done at BioVis core facility at Uppsala University (Sweden). A 5 µl drop of the sample was placed on a Formvar- and carbon coated 200-mesh copper grid. The excess solution was immediately removed by blotting with filter paper. The sample was then washed on three consecutive drops of MQ water followed by two drops of 2% uranyl acetate. Excess of uranyl acetate was removed by blotting on filter paper. Dried grids were examined by Tecnai™ G2 Spirit BioTwin transmission electron microscope (Thermo Fisher/FEI) at 80 kV with an ORIUS SC200 CCD camera and Gatan Digital Micrograph software (both from Gatan Inc.).

### Statistical analysis

The non-linear regression, sigmoidal, 4PL, X is log (concentration) model in GraphPad Prism v.9.4.1 software was used to interpolate the unknown concentrations of the OmpF-MPER from passages 1, 15, 20, 25 and 30 and the Mann-Whitney nonparametric test was used to perform the comparative analysis. Welch’s t test was employed for the comparative analysis of the mean MPER concentration in EcN-MPER and EcN cell lysates.

## Data Availability

The datasets used and/or analysed during the current study are available from the corresponding author upon reasonable request.

## Abbreviations

ART: anti-retroviral therapy
bNAbs: broadly neutralizing antibodies
Cas9: CRISPR associated protein 9
CFU: colony forming unit
CRISPR: clustered regularly interspaced short spaced palindromic repeats
DNA: deoxyribonucleic acid
EcN: Escherichia coli Nissle 1917
EcN-MPER: recombinant EcN containing mper sequence in the ompF loci
ELISA: Enzyme-linked immunosorbent assay
gp41: glycoprotein 41
HIV: human immunodeficiency virus
MPER: membrane-proximal external region
OMP: outer membrane protein
OmpF: outer membrane porin F
PCR: polymerase chain reaction
