# Correction: Stable expression of HIV-1 MPER extended epitope on the surface of the recombinant probiotic bacteria *Escherichia Coli* Nissle 1917 using CRISPR/Cas9

**DOI:** 10.1186/s12934-024-02347-8

**Published:** 2024-03-06

**Authors:** Nathaniel Ninyio, Katharina Schmitt, Gladys Sergon, Charlotta Nilsson, Sören Andersson, Nikolai Scherbak

**Affiliations:** 1 School of Medical Sciences, Faculty of Medicine and Health, Örebro University, Örebro, Sweden; 2 School of Science and Technology, Life Science Center, Örebro University, Örebro, Sweden; 3 Division of Clinical Microbiology, Department of Laboratory Medicine, Karolinska Institutet, Stockholm, Sweden; 4 Department of Microbiology, Public Health Agency of Sweden, Solna, Sweden; 5 Department of Public Health Analysis and Data Management, Unit for Vaccination Programmes, Public Health Agency of Sweden, Solna, Sweden; 6 Institute of Virology, Saarland University Medical Center, 66421 Homburg, Germany

**Microbial Cell Factories (2024) 23:39**

10.1186/s12934-023-02290-0

In this article the author name Katharina Schmitt was incorrectly written as Katherina Schmitt. The original article has been corrected.

